# Reconstruction of Upper Third Ear Defects: Utility of a Limited Tanzer Reduction

**DOI:** 10.29252/wjps.9.2.179

**Published:** 2020-05

**Authors:** Juan M. Colazo, Angel F. Farinas, Vanessa Leonhard, Al Valmadrid, Christodoulos Kaoutzanis, Wesley P. Thayer

**Affiliations:** 1 Vanderbilt University, Medical Scientist Training Program, Nashville, TN, USA;; 2 Vanderbilt University, School of Medicine, Nashville, TN, USA;; 3 Vanderbilt University, Department of Biomedical Engineering, Nashville, TN, USA;; 4 Oklahoma University, Section of Plastic Surgery, Oklahoma City, OK, USA;; 5 University of Washington, Division of Plastic Surgery, Seattle, WA, USA;; 6 Vanderbilt University Medical Center, Department of Plastic Surgery, Nashville, TN, USA;; 7 UC Health University of Colorado Hospital, Division of Plastic and Reconstructive Surgery, Aurora, CO, USA

**Keywords:** Auricular defect, Tanzer, Otoplasty, Auricular flap, Conchal cartilage

## Abstract

**BACKGROUND:**

Large ear defects (>3 cm) present a significant reconstructive challenge and often require extensive operations, which can lead to donor-site morbidity and contour abnormalities. Through our case series, we propose a limited Tanzer reduction, a novel modification of the well-recognized Tanzer technique, as a potential reconstructive option for traumatic and oncologic upper third ear defects.

**METHODS:**

We retrospectively reviewed patients who underwent planned ear reconstruction for large ear defects (>3 cm) at a university center by a single surgeon (WPT) over a five-year period. Demographics, complications, and need for revision surgery were recorded. A satisfaction survey was also completed.

**RESULTS:**

Five patients met our inclusion criteria as they underwent ear reconstruction with the limited Tanzer reduction. All reconstructions followed oncologic resection for cutaneous malignancy. The mean follow-up was 760.2 days. No complications were encountered, and no revisions were required. All cases had good aesthetic outcomes. The satisfaction survey revealed no self-image distortion or social obstacles following the reconstruction.

**CONCLUSION:**

The proposed limited Tanzer reduction technique was shown to be a safe, viable, functionally and aesthetically pleasing option for the reconstruction of large defects of the ear and thus should be part of the armamentarium of the reconstructive surgeon.

## INTRODUCTION

Reconstruction of the head and neck requires recreation of its three-dimensional shape and projection; meanwhile, preserving the local blood supply. With regards to the ear, reconstructive planning is guided by the size and depth of the defect.^1^^,^^2^ Small deficits, up to 1.5 cm, may be closed primarily with a wedge resection and simple closure. In order to reconstruct larger areas up to 3 cm, chondrocutaneous advancement flaps, local rotational flaps, or composite grafts are typically used.^3^

These options allow for coverage and reestablishment of basic ear dimensions; however, the smaller anatomic details within the ear may be lost post-operatively. Large defects, greater than 3 cm, present a unique challenge for surgeons and require more extensive reconstructive techniques, such as local flaps, which have significant potential for donor-site morbidity and contour loss. The temporoparietal fascia flap is commonly used, when posterior skin quality is not optimal, and is a common approach in the repair of burn injuries.^1^^,^^2^^,^^4^^,^^5^

The Tanzer reduction procedure was initially described to address the cosmetic issues associated with the constricted ear.^6^ This procedure includes removal of a portion of the helix to allow the surgeon to create a more aesthetic shape during closure.^7^ The proposed incision size can be similar to defects created following traumatic injuries and oncologic resections, which was the main basis of designing a modified technique. The aim of this study was to describe and discuss the proposed limited Tanzer reduction, which included an ipsilateral cartilage graft and a postauricular flap. This technique was performed in a series of patients requiring reconstruction of large ear defects after the surgical resection of cutaneous malignancies.

## MATERIALS AND METHODS

A retrospective review of all head and neck reconstruction cases for oncologic and traumatic defects at our institution over a five-year period was performed. Out of 622 cases involving skin lesions of the head and neck, five patients were identified who underwent ear reconstruction using the limited Tanzer reduction technique by the senior author (WPT), to best address their individual defect. Representative cases are described below. The medical records of the five patients were reviewed for postoperative complications and reoperations. All patients were asked to complete a satisfaction survey at their last follow-up visit. The study was approved by the Institutional Review Board (IRB) of Vanderbilt University Medical Center (Study Number 172155). 

**Fig. 1:**
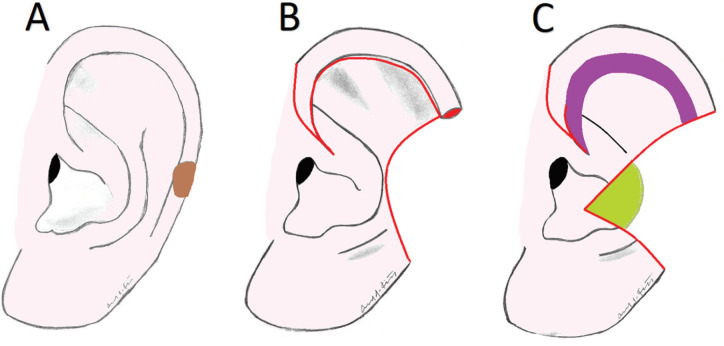
Generalizable operative scheme (Case 1).** A. **Mass at the junction of the upper and middle ear (represented in brown). **B.** Mohs resection with planned superior Antia-Buch chondrocutaneous advancement flap (red outline). **C.** Area of dissection of upper middle ear (represented in purple), and area of exposed conchal cartilage (represented in green).

**Fig. 2:**
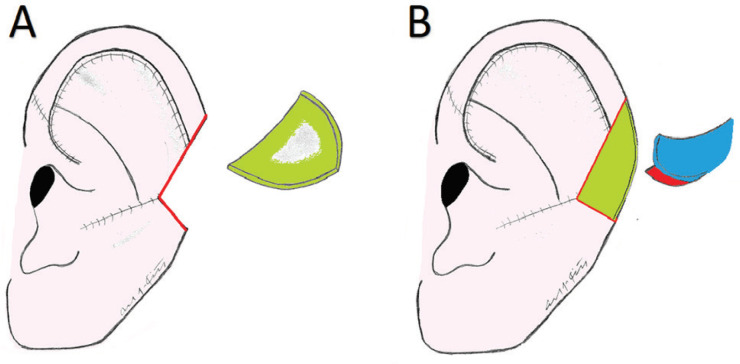
Generalizable Choncal Cartilage Harvest and Flap Creation (Case 1). **A.** Closure of the central ear defect with advancement of the superior Antia-Buch flap (with a V-Y advancement component superomedially) with harvesting of the previously exposed conchal cartilage (represented in green). Note the residual upper-middle ear helical defect. **B.** Conchal free graft used as a framework to close the helical gap, with creation of a posterio-superior rotational advancement flap (represented in blue).

Among cases, case 1 was a 66-year-old female with a past medical history of multiple previous basal cell carcinoma who underwent Mohs resection of a basal cell carcinoma on her left ear (Figure 1A) and presented two days later for reconstruction. The resultant defect involved 60% of the ear, mostly the upper and middle thirds (Figure 1B). A combined approach using a limited Tanzer reduction and Antia-Buch flap was planned. First, the superior Antia-Buch flap was undertaken by resecting a portion of anterior skin and cartilage (Figure 1C). Then, proceeding with resection of a portion of the conchal cartilage and eliminating the defect with interrupted 5-0 Vicryl sutures (Ethicon, Sommersville NJ) and 5-0 chromic sutures (Ethicon, Sommersville NJ) (Figure 2A) was conducted. 

**Fig. 3:**
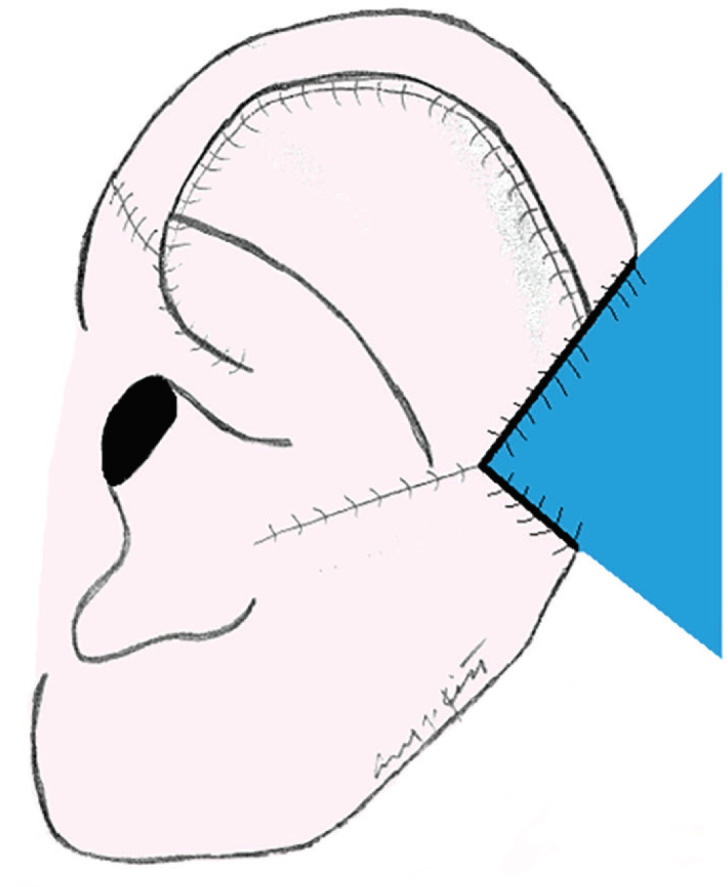
Generalizable Flap Coverage of Helical Defect (Case 1). Posterio-superior flap is inset to bridge the remaining helical defect (represented in blue).

The cartilage was then inverted and used to reconstruct the remaining helical defect. A 6×5 cm rotational advancement postauricular (posterio-superior) flap was then used to cover the free conchal cartilage graft (Figure 2B). The donor site was closed with interrupted 5-0 Vicryl (Ethicon, Sommersville NJ) and the remaining layers with fast absorbing 5-0 chromic gut suture (Ethicon, Sommersville NJ) (Figure 3). The patient was followed up in clinic at one week and one-month post-reconstruction. No complications were noted. The post-reconstruction satisfaction survey was completed 11 months post-operatively (Table 1).

**Table 1:** Post-reconstructive satisfaction survey

**Variable**	**Case 1**	**Case 2**	**Case 3**	**Case 4**	**Case 5**
Do you like the appearance of your ear?	Yes	Yes	Yes	Yes	Yes
Does the appearance of your ear bother you?	No	No	No	No	No
Do you notice a difference in the size of your ear since your surgery?	No	Yes	Yes	No	No
If so, does the difference in the size of your ear bother you?	NR	No	No	NR	NR
Do people comment on the size of your ears?	No	No	No	No	No
Do people notice that you have had surgery on your ear?	No	Yes	No	Yes	No
If so, do people noticing you have had surgery on your ear bother you?	NR	No	NR	No	NR
Overall, are you satisfied with your ear post-operatively?	Yes	Yes	Yes	Yes	Yes

*NR=No response recorded

**Fig. 4:**
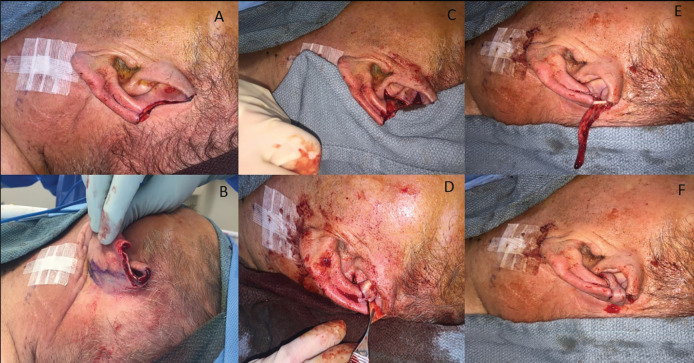
Limited Tanzer Reduction intra-operative sequence (Case 2).** A.** Residual upper third ear defect after Mohs surgical resection. **B. **Posterior view of the defect. **C.** Rotational flap surgically fashioned and choncal graft harvested. **D.** Conchal graft inset (held by Adson forceps). **E.** Tanzer flap banner created to cover the remaining helical defect**. F.** Final surgical coverage of the helical defect

Case 2 was a 70-year-old male with a past medical history of multiple non-malignant skin lesions presented with a confirmed T3a superficial spreading melanoma of the left superior helix without ulceration. Based on a Breslow depth of 3.5 mm with a positive deep margin, a full-thickness resection was performed with a 2 cm circumferential margin, including the skin and cartilage of the ear. The remaining defect involved approximately one-third of the ear (Figures 4A and 4B). The limited Tanzer reduction began with removal of the superior and inferior portions of the scapha as well as part of the conchal bowl (Figure 4C). The detached portion of the conchal bowl was then reshaped and used to reconstruct the helix (Figure 4D). 

**Fig. 5:**
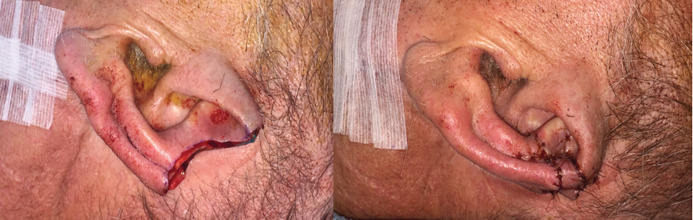
Upper third ear defect before (Left) and after Limited Tanzer Reduction (Right) (Case 2).

**Fig. 6:**
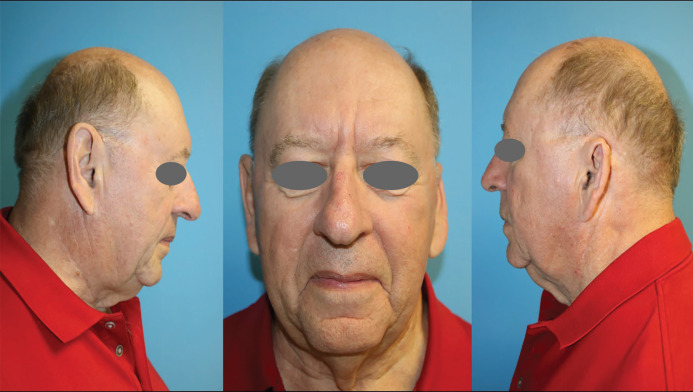
Images taken at a post-operative visit at 11 months (Case 2). Unaltered right ear. **A.** Slightly reduced height of left ear noted when compared to the unaltered right ear. **B.** Surgically reconstructed left ear; viable flap with slight loss of tissue mass in the upper third of the ear

**Fig. 7:**
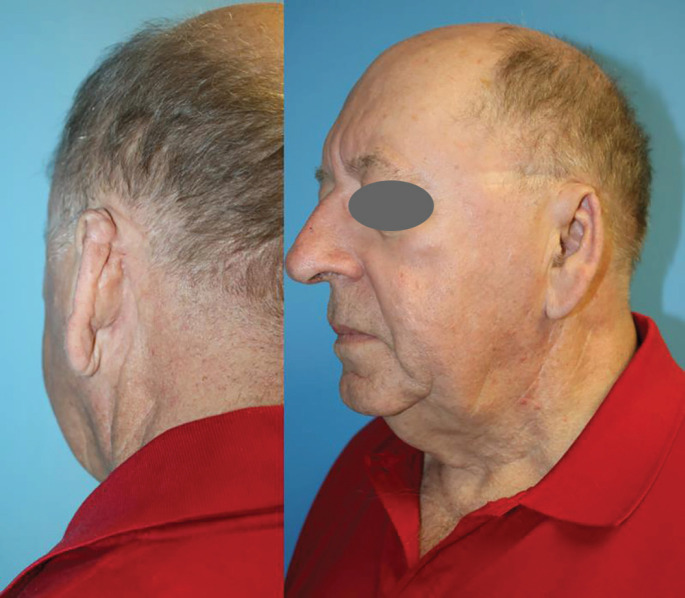
Posterior (Left) and oblique view (Right) of the reconstructed left ear (Case 2). A successful functional outcome can be acclaimed, especially with patients’ use of glasses (noted by skin marks caused by typical use of glasses).

A local random-pattern flap based superiorly off the postauricular crease was then designed and rotated to cover the conchal graft (Figure 4E). After hemostasis was obtained, the cartilage reconstructions were anchored in place with interrupted 5-0 Vicryl sutures (Ethicon, Sommersville NJ) and the remaining layers were closed with 5-0 chromic sutures (Ethicon, Sommersville NJ) (Figure 5). The patient was followed up at one week and one-month post-operatively. No complications were noted. The patient was seen again in clinic at eleven months post-operatively, where images were taken (Figure 6 and 7) and the satisfaction survey was administered (Table 1).

## RESULTS

A total of 5 patients were treated with the limited Tanzer reduction technique including two females and three males with an average age of 66 years. Patients had an American Society of Anesthesiologists Score between II and III. All reconstructions were performed under general anesthesia. All patients were reconstructed after excision of a malignant lesion in the upper third of the ear. Basal cell carcinoma was the most common type of lesion in this region, followed by melanoma. In most cases, a rotational flap was used to cover the final defect; one patient required a split thickness skin graft for additional coverage. 

Ipsilateral conchal cartilage was used in four out of the five patients. The average duration from diagnosis to resection was 55.6 days. Four out of the five cases were performed as immediate reconstructions, while one case was a delayed reconstruction and was closed two days after the initial resection (Case 1). No complications were encountered, and no revisions were required. Patients were given a post-operative survey in-person at a follow-up appointment or contacted via telephone (Table 1). 

The period between surgery and determination of satisfaction ranged from 30 to 1835 days, with a mean of 760.2 days. All five patients were satisfied with the appearance of their ear. Three of the five patients did not notice a size difference as compared to their previous ear and the remaining two did not mind the discrepancy. Two patients had social feedback about their repair with no self-image or psychological consequences. Ultimately, all five of the patients reported that they were pleased with their reconstructive outcome.

## DISCUSSION

Successful repair of auricle defects requires framework and adequate vascularized tissue coverage.^8^ Partial ear reconstructions have been performed as early as 600 BC.^9^ For restoration of the structural foundation, Harold Gilles first proposed the use of autologous cartilage, a method that is still recommended for larger congenital and traumatic defects.^4^ In these cases, the strong, sizeable, framework available through the use of costal cartilage (from the ribcage) outweighs the potential donor site complications, which includes infection, pneumothorax, and chest wall deformities.^4^^,^^10^

These cartilaginous struts are essential in rebuilding and maintaining the intricate dimensions of the ear.^8^ For smaller defects, structural support is equally vital. The conchal cartilage (from the ear) is frequently used as a composite graft due to its accessibility, flexibility, and minimal donor site morbidity.^1^^,^^8^ Additionally, the concha, like the triangular fossa and the scapha’s cartilages, may be removed without affecting the auricular framework.^1^ In most of our case series (four out of the five patients), the ipsilateral concha was used to form the framework of the reconstruction. At follow-up, all patients were satisfied with their structural outcome.

Tissue coverage, which is the second component, is determined primarily by size and location of the deficit. Auricular defects can be classified into an upper, a middle, and a lower third.^1^ Traumatic injuries such as bites, motor vehicle collisions, and burns most commonly affect the upper third.^4^^,^^8^ The upper third is the most prevalent area for tumors due to its propensity for increased sun exposure.^1^^,^^2^ Our proposed reconstructive technique provided a novel reconstructive option for the commonly affected upper third of the ear. 

Techniques for small and medium defects of the antihelix and helix have well-defined surgical options involving primary closure, tissue advancement, or the use of composite grafts. The Tanzer technique builds upon these concepts and incorporates a star-like resection, where two small triangles are added on each side to aid in closure. This allows for coverage of the defect, as well as adequate structural preservation.^1^^,^^7^ The coverage was completed using classic helical advancement or posterior auricular skin flaps, as both are easily accessible and mobile.^1^^,^^4^

The posterior auricular flap is based on the postauricular artery and has strong, reproducible results.^1^^,^^4^

Four cases in this series were closed with a posterior flap, while one also required skin grafting. It must be noted that this technique can also be used in conjunction with other local flap options, such as in one of our cases, where we used it with an Antia-Buch flap (Case 1). Previous studies have demonstrated that patients struggle more with acquired lesions than with congenital defects. In oncologic resections, therefore, functional and aesthetic outcomes are a priority.^5^ In high-risk or poor surgical candidates, defects can be camouflaged with a prosthesis, such as porous polyethylene implants, to provide an adequate and simple repair without extensive surgery.^1^^,^^2^^,^^4^

Consideration of the postoperative goals, health status, and defect type allows surgical teams to individualize each patient’s operative plan appropriately.^11^ Several aesthetic factors must be considered in these repairs, including symmetry, awareness of overcorrection, and maintenance of anatomical landmarks and contour.^11^

Functional outcomes, like wearing glasses (Case 2) or hearing aids, are equally important and are paramount in successfully meeting patient expectations.^2^^,^^3^ The use of the stellate incisions proposed by Tanzer combined with classic reconstructive techniques allowed us to provide coverage, recreate the ear structure, and address each of these factors. Complications associated with ear reconstruction occur in approximately 8% of cases and include hematoma, infection, and skin necrosis with cartilage exposure.^11^^,^^12^

Late complications such as granulomas, suture extrusion, hypertrophic scarring, keloids, fistulas, and chronic wounds may also develop.^11^^,^^13^ In this case series, neither immediate nor late complications were observed. The limitations of this study originated from variations in the care of each patient and the relatively small sample size of this case series. This individualized procedure, based on the dimensions and structures involved, varied slightly between all cases and can alter the reported outcomes of patients. 

While the ability to modify steps of the Tanzer reduction to reconstruct unique defects may be advantageous to the surgical team, these variations introduce potential for discrepancies in satisfaction surveys. The outcomes reported here are based upon retrospective subjective surveys, which intrinsically introduce recall bias into the results (Table 1). Finally, the timeline for postoperative satisfaction surveys varied, providing a temporal range of feedback, but this method does not determine how these reconstructions will develop with time.

The Tanzer reduction procedure, originally described to treat congenital defects, can be safely applied to reconstruct large auricular defects using ipsilateral concha cartilage and a posterior auricular flap, known as a limited Tanzer reduction. It can also be used in conjunction with other local ear flaps to reduce the morbidity from distant areas. The limited Tanzer reduction procedure proposed here provided excellent functional and aesthetic outcomes in our case series of five patients with post-oncologic defects, but could be utilized in and varied for multiple different defect types.
